# Bronchogenic cyst with multiple complications

**DOI:** 10.2349/biij.3.4.e42

**Published:** 2007-10-01

**Authors:** G Marshall, C Cheah, NP Lenzo

**Affiliations:** 1 Fremantle Hospital, Fremantle, Australia; 2 Department of General Medicine, Fremantle Hospital, Fremantle, Australia

**Keywords:** Bronchogenic cyst, atrial fibrillation, pleural effusion, pericardial effusion, computed tomography

## Abstract

Bronchogenic cysts are a rare type of mediastinal mass thought to arise from abnormal budding of the embryologic foregut. This paper presents a rare case of a 32-year-old male who developed multiple serious complications from a bronchial cyst. This rare presentation is discussed and the role of CT and MR imaging in making the diagnosis is highlighted.

## INTRODUCTION

Bronchogenic cysts are a rare cause of mediastinal mass [1,2]. The embryologic foregut differentiates into oesophagus and trachea during embryogenesis. Bronchogenic cysts occur along the differentiating pathway of the trachea and bronchial tree, and are thought to represent abnormal budding of foregut tissue. The thick walls of bronchogenic cysts may contain columnar ciliated epithelium, cartilage and mucinous glands. They are most often found in the mediastinum or lung, and sometimes in the neck [1]. Bronchogenic cysts are typically asymptomatic, with many detected incidentally. However, complications do occur. Resection of even the asymptomatic cyst is recommended because of the possibility of complications and the difficulty of operating on complicated cysts [2,3].

## CASE

**Figure 1:**
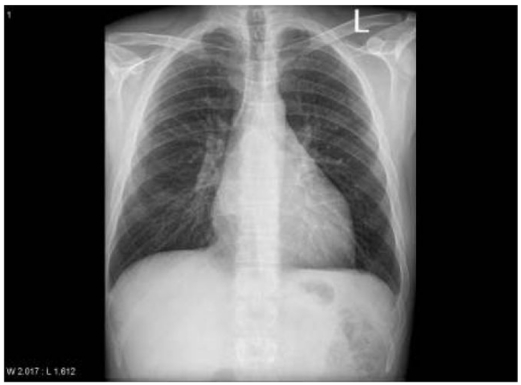
CXR with splaying of the carina.

**Figure 2:**
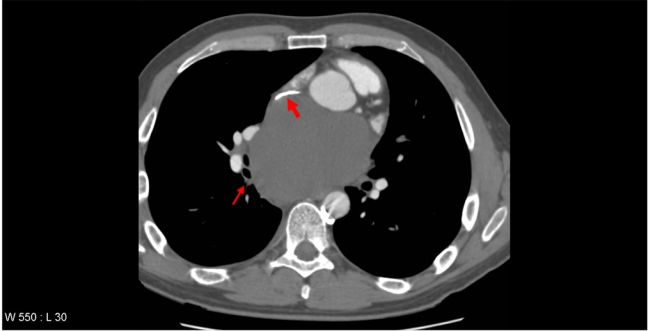
Computed tomography imaging of chest, with intravenous contrast showing superior vena cava compression (small arrow) and right inferior pulmonary vein compression and thrombus (large arrow).

**Figure 3:**
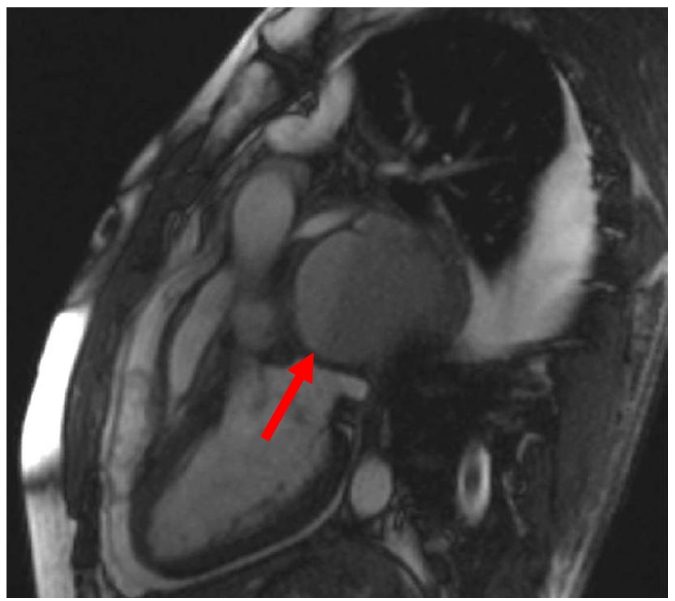
Cardiac MRI with large cyst (arrow - internal fluid level)

**Figure 4:**
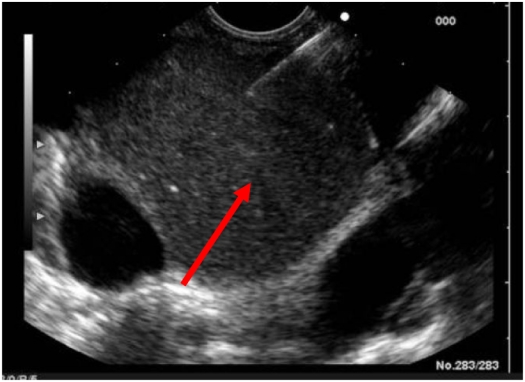
Endoscopic transoesophageal ultrasound guided fine needle aspiration (arrow - hyperechoic particles within cyst)

An otherwise fit 32-year-old male presented with a dull central chest pain, which gradually worsened over several days with associated night sweats. Chest radiography (CXR) showed a mediastinal mass (Figure 1). CT with contrast showed a 10 cm posterior mediastinal mass inferior to the carina and adjacent to the left pleura, left atrium, pulmonary artery and oesophagus. There was marked mass effect with left atrial and superior vena cava (SVC) compression, right inferior pulmonary vein congestion and thrombus (Figure 2). Clinical assessment did not reveal evidence of superior vena cava obstruction or haemodynamic compromise. Given the history of sweats, and age of the patient, a provisional working diagnosis of mediastinal lymphoma was made and fine needle aspiration was pursued. Transthoracic echocardiography demonstrated the mass as separate from the left atrium, however further comment on likely diagnosis was not possible from the views obtained. Cardiac magnetic resonance imaging (MRI) 48 hours after presentation showed a smooth walled structure not communicating with the heart, oesophagus or bronchus, with the additional development of pericardial and pleural effusions. High-dose dexamethasone was commenced for treatment of presumed lymphoma until confirmatory tissue could be obtained. The patient subsequently developed symptoms and ECG changes that was consistent with pericarditis and runs of atrial fibrillation (AF). An endoscopic transoesophageal ultrasound-guided fine needle aspirate (EUS-FNA) was performed as the mass was considered too technically difficult to access by percutaneous CT-guided FNA. The images at EUS-FNA showed a fluid-filled cyst with hypoechoic particles (Figure 3). Frank pus was aspirated from the cyst. *Streptococcus salivarius* and *Streptococcus milleri* was cultured from the aspirate and a diagnosis of an infected cyst was made. Retrospective and detailed review of all MR images yielded one sagittal view demonstrating a fluid level within the cyst. (Figure 4) This had not been visible on CT or transthoracic ultrasonography. IV amiodarone (for AF) and anticoagulation (for pulmonary vein thrombus) was commenced and cardioversion occurred within 12 hours of procedure. Steroids were ceased and IV ticarcillin and potasssium clavunate (Timentin, GSK) was commenced. The patient was referred to Cardiothoracic Surgeons for further management.

Upon thoracotomy the mass was identified, but could not be removed because of its adherence to oesophagus, pericardium and pleura. It was thus marsupialised into the oesophagus. A nearby enlarged mediastinal lymph node was removed for histopathologic examination. Histopathology revealed a reactive lymph node and material taken from the cyst wall displayed pseudostratified ciliated respiratory type epithelium and glandular structures. No cartilaginous material was seen in the excised section of the cyst wall. There was evidence of inflammatory infiltrates in the sample. Post-operatively, the patient was anti-coagulated (due to the pre-operative presence of thrombus on the CT) and was discharged home once recovered 14 days after admission.

## DISCUSSION

Serious complications from bronchogenic cysts are rare, but can include SVC syndrome, tracheal compression, pneumothorax, pleurisy and pneumonia [4]. In a series of 68 paediatric and adult cases, 58% of patients were symptomatic with pain (n=15), dyspnea (n=8), respiratory infection (n=7), wheezing (n=5), cough (n=5), dysphagia (n=1), pneumothorax (n=1). The cyst diameters in this study were 1.3 cm to 11 cm, with a mean of 4.8 cm [5]. Atrial fibrillation secondary to bronchogenic cyst has been previously described. Electro-physiological studies in a patient with bronchogenic cyst and right inferior pulmonary vein impingement showed atrial ectopic foci in the region of the distended vein [6]. Stretching of the pulmonary vein is known to lead to abnormal firing of myocardiocytes. This was thought to produce atrial fibrillation in the reported case [6]. CT is commonly used in assessing mediastinal masses. While the anatomy of the mediastinal mass is easily seen, the modality is limited in its ability to diagnose bronchogenic cysts of high density. The attenuation of the cyst’s contents can vary from that of water to soft-tissue [5,7]. The value of attenuation can be as high as 100 Hounsfield units if the cyst contains protein or calcium oxalate [7]. In this case, the combination of MRI, which revealed a fluid level and better defined the anatomical relationships of the mass, together with transoesophageal ultrasound, which characterised the internal structure of the mass and allowed diagnostic aspirate, were critical to management.

## CONCLUSION

Bronchogenic cyst of the mediastinum can present acutely with several complications. In this case, the mass effect and the presence of inflammation were likely related to the development of complications. The multiple complications observed in this patient were: atrial fibrillation, pericardial and pleural effusions, and pulmonary vein thrombosis. There was also radiologic evidence of left atrial compression and SVC obstruction without obvious significant clinical signs or symptoms. This case is of interest due to the multiple complications and the diagnostic dilemma this case presented. The diagnosis may be easily missed on CT and transthoracic ultrasound. MRI and endoscopic transoesophageal ultrasonography were in this case helpful in accurate characterisation of the mass.
